# Different approaches and their consequences for addressing the occupational health and safety of young workers: A systematic narrative literature review

**DOI:** 10.1177/10519815251319240

**Published:** 2025-04-24

**Authors:** Gilles Descloux, Mathilde Romanens, Nadia Lamamra, Barbara Duc

**Affiliations:** 1 The Swiss Federal University for Vocational Education and Training (SFUVET), Renens, Switzerland

**Keywords:** vocational education, mental health, physical health, social working conditions, determinants of health, adolescent health

## Abstract

**Background:**

The last twenty years, studies have examined ‘young workers’ occupational health and safety (OHS). Depending on the discipline, approach and methodology, they address youth OHS in different ways. This systematic narrative review aims to provide a deeper understanding of this research landscape.

**Objective:**

The first objective is to provide a systematic review of the literature on OHS of ‘young workers’. It consists of reviewing the literature by disciplines, approaches, methods, data and factors, and of focusing on studies that address the contextual and social aspects of OHS. The second objective is to consider the specific situation of apprentices.

**Methods:**

Searches were conducted in five scientific databases, supplemented by three resource platforms. The criteria of literature selection were: OHS of ‘young workers’ aged from 15–24; explicit link between work and health; publication between 2005 and 2022 in Europe, North America and Australia. Studies have been categorised.

**Results:**

193 studies were included. The review shows the diversity of the population studied under the same heading and the predominance of medical studies, which favour epidemiological approaches and quantitative data. Other disciplines (e.g., psychology, educational sciences, social sciences), approaches (e.g., organisational, risk perception), data (e.g., interviews, observations) and methods (qualitative, mixed, longitudinal) are marginal. Individual and age-related factors are predominant. The impact of working conditions and social relations on OHS is rarely considered.

**Conclusion:**

Research on OHS through organisational context and social relations, particularly employment status, should be encouraged. This provides a deeper understanding of the constraints faced by ‘young workers’, especially apprentices.

## Introduction

‘Young workers’, whether employed (qualified or not) or in training (dual VET or full time school) (The more precise category to which the mentioned studies refer will be specified wherever possible.), have significantly higher rates of occupational injury than do adult workers whether in the US, Canada, Australia or Europe.^1,2^ They also appear to be more vulnerable to workplace violence and harassment, which are contributing factors for the development of cardiovascular disease, depression, burnout, anxiety, nervousness, reduced job satisfaction and reduced well-being.^1^ Based on these considerations, many studies specifically address the occupational health and safety (OHS) of young people. Some of them emphasize that this population tends to accumulate risk factors in terms of physical and mental health^3,4^ and focus on these various risk factors.^5^ Unlike previous literature reviews^6^ identifying this kind of research, the current paper does not aim to identify the risk factors and their impact on health, but proposes a systematic narrative review^7^ from 2005 to 2022. It aims to gather existing literature in all disciplines that address ‘young workers’ OHS in a systematized manner, then qualitatively interpret knowledge to demonstrate the value of a particular point of view.^8^ In this case, it highlights the literature that emphasises the organisational and social aspects of OHS. The latter makes it possible to apprehend the specific situation of apprentices.

The papers published during this period will also be discussed in the light of a previous literature review conducted by Laberge and Ledoux,^9^ that cover the period from 1994 to 2005. The latter, from which the classification method is inspired, serves as a point of comparison for the evolution of research (themes, approaches, etc.). However, the current paper aims to go beyond a simple comparison, by focusing on certain aspects little studied by Laberge and Ledoux,^9^ such as approaches, disciplines, and methods employed. These central aspects have been considered to as they have consequences regarding the way in which the phenomenon is apprehended. In that perspective, the salient aspects and the blind spots of this literature will be looked at, in order to highlight the breakthroughs enabled by different disciplines and approaches, even those that are still marginal.

First, the methodological section will describe the search criteria and strategies used, as well as the classification method adopted, to select and categorise the papers. Second, the reviewed literature will be presented in the form of a systematised table. The latter will allow a first level of descriptive results, that will highlight the primacy of certain disciplines, approaches, and entry points through which the literature addresses ‘young workers’ OHS. A second section of the results will underline some themes, that seem relevant to us, even if they are not dominant. Last, a discussion will put the results into perspective, in regard to the literature review by Laberge and Ledoux^9^ and will open up new perspectives in terms of disciplines, approaches or methodologies. It will enable to consider more in details the OHS’ issues of ‘young workers’, and especially of apprentices, in order to explore other factors explaining their greater vulnerability with regard to health at work.

## Methods

### Research criteria and strategies

This systematic narrative review considers literature published between 2005 and 2022 (Table 1). This period allows for the discussion of results in light of a previous literature review covering the 1994–2005 period.^9^ The selected studies specifically address the OHS of ‘young workers’, often associated with the 15–24 age group.^1^ This category refers, on the one hand, to the minimum age for entry into employment, generally set at 15,^1^ and, on the other, to a general consensus in the literature on the upper limit, set at 24.^10^ This categorisation presents limitations due to the heterogeneity of the situations to which it refers^10,11^ and the fact that it brings together two categories with distinct realities, as identified by Arnett^12^: adolescents (10–18 years old) and emerging adults (18–25 years old). Therefore, research relating to the broad category of ‘young workers’ was included to avoid excluding studies assimilating apprentices to this broader population. However, papers dealing with young adults (using the keywords ‘young adult’ or ‘young working adult’) or those comparing ‘older workers’ with ‘young workers’ were excluded in order to specifically address the OHS issues of a juvenile population.

**Table 1.** Criteria of inclusion and exclusion of studies.

	Included	Excluded
Publication period	2005 to 2022	
Population (age and status)	Studies referring explicitly to ‘young workers’, whether they are in training (apprentices and vocational students) or employed (skilled or unskilled), aged 15–24 years	Studies in which ‘young workers’, apprentices, and vocational students are part of a larger category of workers and are not specifically analysed
Themes	Explicit link to physical and mental health at work	Child labour, health of young people who are unemployed, on sick leave, or with disabilities
Languages	French, English, and German	Spanish, Italian, and Portuguese
Geographical areas	Europe, North & South America, Canada, and Australia	
Type of work	- Peer-reviewed articles - Official studies and reports (online access)	- Theses - Books and book chapters - Non-peer reviewed articles - Magazine articles - Unpublished reports
Disciplines	All	None

Only articles in which the link between work and health effects is explicit have been retained, covering the effects of work on both mental and physical health and whether these injuries are recognised as ‘occupational diseases’ by insurance companies. Papers dealing with risky practices, such as gambling, alcohol, or tobacco consumption, or individual health practices in the private sphere (e.g., sports) were excluded unless the link with work or training was explicit.

The corpus includes European studies published in French, German, and English (This choice was based on the authors' knowledge of these three languages.), as well as North American and Australian studies. It integrates all disciplinary approaches to broaden the perspective of a research field strongly marked by certain disciplines with their own epistemology and issues.

Queries were carried out between August 2022 and January 2023 across several databases by selecting peer-reviewed scientific articles and expert reports published by research institutes and available online. Papers not appearing in these databases because they used other publication formats (e.g., dissertations or doctoral theses) were not included, nor were articles without empirical data or non-peer-reviewed publications (e.g., certain books, book chapters, and articles in popularisation magazines).

The data were collected by consulting the following scientific databases using a date filter: CISDOC, JSTOR, Cochrane Library, the International Bibliography of the Social Sciences (IBSS), and PubMed. The databases used are internationally recognised and frequently referenced in scientific literature reviews. These databases are complementary in that they focus on OHS (CISDOC), specialise in health sciences (Cochrane Library and PubMed) or social sciences (IBSS), or are multidisciplinary (JSTOR). The electronic resource platforms Cairn and Wiley Online Library, as well as the Google Scholar search engine, were also used as complementary resources.

In addition, the search was conducted using the ‘snowball’ method. The references initially identified were supplemented by those cited in the text or bibliography of the consulted articles (‘backward search’). The databases, platforms, and additional references accessed enabled a more exhaustive range of studies, and the search was carried out to saturation.

Two groups of keywords, generally mentioned in the titles of papers and related to the research topic, were utilised with the Boolean operators ‘AND’ and ‘OR’.^13^ These include ‘health’, ‘occupational health’, ‘safety’, ‘safety at work’, ‘OHS’ AND ‘youngsters’, ‘young workers’, ‘adolescents’, ‘apprentices’, ‘apprenticeship’, ‘vocational high school’, in both French and English (For international dissemination and regardless of the language in which the articles are written, most journals offer keywords and abstracts in English. We therefore felt it was sufficient to run our queries in English and French.). Since a search equation is iteratively constructed based on the noise or silence of the query,^14^ synonyms for these terms were also included. The search using the term ‘adolescent’ appeared relevant, as this designation, mentioned in the titles of relevant papers, includes both young people in vocational training^15,16^ and ‘young workers’.^17,18^

The corpus was built in several stages. An initial extraction of the papers was carried out by reading the abstracts extensively. This first extraction required discussion between the authors to determine the eligibility of certain studies. A consensus-based collective clarification excluded studies dealing with unrelated themes (i.e., health-risk practices with no explicit link to work, child labour, the health of young people with disabilities, unemployed, or on sick leave, etc.). This second screening resulted in the selection of 193 articles (The 193 articles selected for this literature review are listed in Appendix A, including those cited (numbered in brackets). Other articles are referenced according to APA standards (7th edition).).

### Processing method

The 193 articles were reviewed in depth by two of the authors working collaboratively and systematically classified using the iterative grouping technique.^9^ This technique involves systematic descriptive coding (types of articles, content, results), consultation with the research team, reflecting back to the classification categories (disciplines, approaches, methods, themes), sorting collectively according to these categories, and refining into sub-categories (Table 2 & Table 3). The collective work was realised in order to avoid errors.

**Table 2.** Classification categories.

Geographical areas	Europe, North America, and Australia
Population	Status
	Sectors
Type of research	Disciplines
	Approaches
	Data
	Methods
Individual characteristics	Sociodemographic background (age, sex, and socio-economic status)
	Educational and training pathway
	Genetic predispositions, cognitive disabilities, and behaviours
	At-risk consumption and lifestyle
Organisational and contextual factors	Company characteristics and style of management
	Work organisation and job constraints
	Occupational exposures
	Social risks and environment
	Safety devices and training
Physical and mental impairments	Accident
	Pathology
	Psychopathology
Other	COVID-19
	OHS education programs

**Table 3.** Description of the literature (the number of listed articles is shown in brackets).

Geographical areas		Europe (93), North America (71), and Australia (27)
Population	Status	Apprentices (93), young workers (65), vocational students (40)
	Sectors	Not clearly specified (71), construction (63), hairdressing and beauty (30), hotel and catering (24), industries and mines (21), sales occupations (20), health care (16), food trades (15), agricultural professions (14), administration, banking and insurance (13), other sectors of activity (12), logistics and transportation (11), automotive and mechatronics (10)
Type of research	Disciplines	Medicine (industrial/occupational medicine, physical therapy, rehabilitation, public and environmental health, psychiatry) (115), transdisciplinary (37), educational sciences (10), psychology (10), business management and economics (7), ergonomics (7), social sciences (sociology, anthropology, psychodynamics) (5)
	Approaches	Epidemiological or medical (99), organisational (47), other (37), preventive (27), vocational training and educational perspective in OHS (26), supervision (17), evaluation of risk perception (16), socio-structural (16), work activity (9), gender and ethnic studies (7), legal or historical (2)
	Data	Statistics (125), medical and psychometric tests (34), interviews (33), documentary inquiries (18), mixed data (16), observations (14), participative experiences (13)
	Methods	Quantitative (138), among them longitudinal (35), qualitative (35), mixed (19)
Individual characteristics	Sociodemographic background	Sex (98), age (79), socio-economic status (46), ethnic group or immigrant background (19), residence (19), familial background (16), pregnant or have children (2)
	Educational and training pathway	Previous work curriculum like years in apprenticeship program or training experience (33), distance to work (1)
	Genetic predispositions, cognitive disabilities, and behaviours	Motivation, satisfaction (17), atopy (12), previous diseases (10), risk-taking behaviour (9), physical characteristics (weight, height, body mass index) (7), identity (self-esteem and self-perception) (5), learning disabilities (2)
	At-risk consumption and lifestyle	Tobacco consumption (31), alcohol consumption (29), drug consumption (20), physical activity (16), amount of sleep or feeling tired (10), food consumption (6), sexual health (2), gambling (1)
Organisational and contextual factors	Company characteristics and style of management	Company characteristics (size of company, type of production, etc.) (25), style of management (6)
	Work organisation and job constraints	Work social support (53), physical work demand and load-bearing (43), work schedule (35), division of work (31), psychological work demand (27), part-time job or job combination (24), employment status (type of contract, black job, informal job, etc.) (23), job insecurity (21), work decision latitude (16), trade-union member or contact (9) school-based or on-the-job training (3)
	Occupational exposures	Chemical agent (39), machine, tools (injuries, modalities of usual usage, dangerous devices knowledge) (33), vibration, noise, or dust (19), temperature and weather (11), work on the screen (4), biological agent or radiation (2)
	Social risks and environment	Harassment like bullying, mobbing, isolation, verbal abuse by supervisor or coworkers (29), customer aggression like pressure, kidnapping, verbal abuse (15), gender or racial discrimination like isolation, sexual harassment (14)
	Safety devices and safety training	Safety training at work or training centre (45), equipment (use of special equipment), safety environment, workplace design (44),
Physical and mental impairments	Accident	Not specified injuries (32), sprain, strain, hernia (25), accident with or without work interruption (16), burns, heat chemical burn, or electrocution (16), medically intended work injuries (15), fall or slipping (12), abrasion, contusion, bite, crushing (11), broken bones, fractures (11), fatal injuries (9), past or repeated injuries (9), amputation (7), asphyxiation (1)
	Pathology	Skin hazards (35), musculoskeletal disorders (MSDs) (23), shoulder pain, back pain (19), respiratory disorders like asthma, wheezing, etc. (17), sleep disorders (11), any physical disorder, not specified (9), sneezing, runny or rhinitis (8), symptoms like fever, flu-like, headaches, etc. (8), hearing impairment (7), carpal tunnel syndrome (CTS) (6), headache (5), lung diseases (5), others impairments like blood pressure, blood glucose, heart rate, etc. (3), cancer (2), cardiovascular disease (2), eating disorders (1)
	Psychopathology	Well-being (WHO-5 Well-Being Scale) (31), stress (23), psychological distress, anxiety (GAD-7, ASQ test) (19), depression (MDD, CES-D test, PHQ-9, etc.) (12), tiredness (10), concentrating disorder (7), phobia, fear, sadness (6), not specified (5), suicidal ideation or attempts (5), consulting specialist for mental troubles like therapists, social workers, etc. (3), ethical suffering, sense of work (4), mental toughness (MTQ48 test) or psychological resources (3), burnout (1)
Other	COVID-19	COVID-19 symptoms, COVID-19 period effect, etc. (3)
	OHS Education Programs	Content, Transmission Obstacles, Programs Content Appropriation (26)

Table legend: generally, the chosen nomenclature used aligns with that found in the literature, particularly concerning the qualification of physical and mental injuries. A study may cover one or more occupations, grouped here by sectors of activity. Articles that do not specify the examined occupations are classified in the ‘not clearly specified’ category. The ‘other work sectors’ category includes other areas of activity that are occasionally examined.

The type of research was classified as follows. a) By disciplines: This classification is based on the authors’ and journals’ disciplines. The categories of disciplines are formed by epistemological affinities (e.g., ergonomics researches have specific concepts and theoretical frameworks i.e., the study of work sequences of gestures). The ‘transdisciplinary’ category is applied when the first two authors come from different disciplines, without necessarily implying an interdisciplinary or multidisciplinary approach. b) By approaches: The socio-structural approach refers to work that examines the socio-economic determinants of exposure to disease, accidents, or access to health (including level of education, parents’ socio-economic situation, and school dropouts); the work activity approach refers to studies of actual everyday work (gestures, interactions, rhythm, etc.). c) By type of data: this involved data collected through questionnaires (statistics), interviews, or observations, and in addition ‘medical tests’ and ‘participative experiences’ specific to the medical or prevention fields.

The categories used are partly based on those of Laberge and Ledoux^9^: demographic, personal, occupational, organisational, temporal, and operational factors. The categories retained as they stand are ‘individual characteristics’ (sex, ethnic origin, etc.) and ‘organisational and contextual factors’. The ‘operational factors’ category was discarded because, according to our classification, it falls under several sub-categories of ‘organisational and contextual factors’. A rearrangement of their categories was carried out, with ‘occupational health and safety risks’ referring here to ‘physical and mental impairments’ and the category of ‘personal factors’ (habits, family antecedent) being considered a subcategory of ‘individual characteristics’.

Finally, the categories of ‘temporal constraints’ (working time, schedules, etc.) and ‘psychosocial factors’ (decision latitude, social support, etc.) identified by the above-mentioned authors are assimilated in our review to ‘organisational and contextual factors’. These refer to elements such as the characteristics of the company and types of management; work organisation and job constraints; occupational exposures; social risks; and safety devices and training. Finally, other categories have been added to situate the various contributions to research on the OHS of ‘young workers’: ‘geographical area’, ‘population’, ‘type of research’, and ‘COVID-19’.

## Results

The results will be structured as follow: a first section will propose descriptive results based on a systematic narrative review, and a second section will underline some themes, that seem relevant to us, because they highlight the social and organisational context, in which OHS is embedded.

### Overview of the literature

**Figure 1.**
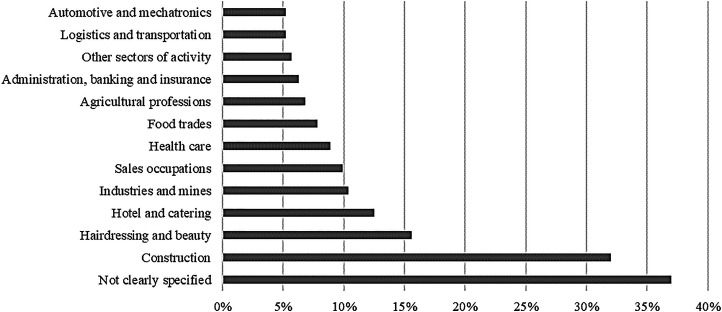
Occupational sectors addressed in the literature.^7^.

The various types of study examined in this systematic narrative review appear in Table 3. Regarding the analysed population, almost half the studies specifically address young people in training, whether they are apprentices (N = 93) or vocational students (N = 40). However, a third of the literature, particularly in the US, carries out surveys of ‘young workers’ (N = 65) without specifying whether they are young people in training or skilled or unskilled ‘young workers’. One third of the studies (N = 71) examined the OHS of ‘young workers’ without focusing on a particular occupation. However, the construction, hairdressing and beauty, hotel and catering, industries and mines, and sales occupation sectors are well explored (Figure 1) in contrast to the administration, banking, and insurance sector, logistics and transportation sector, and automotive and mechatronics sector.

Analysis of the type of research shows that a significant proportion (58%) comes from the disciplines of medicine (N = 115). This literature is generally based on a traditional epidemiological approach (statistical correlations between ‘exposure’ or ‘risk factors’ and ‘health impairment’). The importance of this type of study is partly due to the fact that OHS has historically been managed by an occupational medicine mainly focused on individual (biological, genetic) factors, while socio-economic factors have received less attention.^19^ Psychology (N = 10), educational sciences (N = 10), business management and economics (N = 7), ergonomics (N = 7), and social sciences (N = 5) account for only one-tenth of the literature (22%). In direct relation to this finding, the preferred approaches are mainly (52%) medical or epidemiological (N = 99). The organisational approach is half (25%) as important (N = 47). Other approaches are also scarce (18%): evaluation of risk perception (N = 16), work activity (N = 9), gender and ethnic studies (N = 7), legal or historical (N = 2).

Disciplines and approaches influence the types of data collected (statistics, medical, and psychometric tests (WHO-5 Well-Being Scale, SF36 test, K10 scale, Short Form-12 Health Survey SF-12, GHQ-28, etc.)) and methods, which are mainly (72%) quantitative (N = 138). Interviews (N = 33) or mixed data (N = 16) are also employed, but less frequently (25%). A few months after the start of vocational training or work, longitudinal approaches (N = 35) are used to measure the physical impairment caused by exposure to toxic substances or repetitive movements or to measure the effectiveness of a prevention programme.

The literature identifies two types of risk factors. On the one hand, individual factors, i.e., individual characteristics (age, sex, socio-economic status, ethnic group, etc.), educational and training pathway, genetic predispositions, cognitive disabilities and behaviours, and at-risk consumption and lifestyle (alcohol or drug consumption, physical activity, etc.) are taken into consideration. On the other hand, organisational and contextual factors such as the company characteristics (size of company, type of production, etc.), work organisation and job constraints, occupational exposures (chemicals, tools, dust, etc.), and social risks and environment (harassment by supervisor or coworkers, customer aggression, etc.) are also considered.

Three types of health impairments are identified (Table 4). One third of the literature (N = 61) addresses workplace accidents (fractures, burns, etc.), another third focuses on mental health impairment (N = 64), and the remaining studies examine physical health impairment (N = 76). One-eighth of all these works consider both accidents and illnesses (N = 26), while fewer than a one-tenth explore illnesses and mental health impairment (N = 15), or all three types combined (N = 15).

**Table 4.** Literature sorted by type of health impairment.

Articles	Physical health impairment	Accidents	Mental health impairment	Physical health impairment and accidents	Physical and mental health impairment	Accidents and mental health impairment	All three
N = 191	76	61	64	26	15	5	15
Percent	39.8	31.9	33.5	13.6	7.9	2.6	7.9

Physical health impairments concern occupational illnesses, mostly of them due to occupational exposures (chemicals, biological agents, heat, etc.), repeated movements and postures, and physical stress. The focus is on skin hazards (N = 35), MSDs (N = 23), chronic joint pain (shoulder and back pain) (N = 19), respiratory disorders (N = 17), and sleep disorders (N = 11). Mental health is addressed in terms of the general state of well-being (N = 31), stress level (N = 23), psychological distress and anxiety (N = 19), or depression (N = 12). Only one study specifically addresses burnout. Accidents mostly refer to any types of not specified injuries (N = 32), to sprains, strains, hernias or wounds (N = 25), to burns or electrocutions (N = 16), to accidents in relation with or without work interruption (N = 16) or injuries requiring medical intervention (N = 15), and to falls or slipping (N = 14). Some studies address fatal injuries (N = 9) and past or repeated injuries (N = 9).

Other themes are sometimes addressed, in particular OHS education programs, through their content, transmission obstacles, or, more rarely, the ways in which this knowledge is appropriated. This type of study has quite developed over the last twenty years (N = 26) insofar as Laberge and Ledoux (2011) identify eleven studies dealing with organisational safety practice and OHS-problem prevention for young people. What emerges is the variability in OHS training in terms of content, duration, and degree of formalisation. Generally, OHS aspects appear to be underdeveloped or even absent from programs and limited in scope. The literature does, however, consider that OHS training has a significant protective potential for young people. However, contradictions often exist between the health practices and knowledge provided by trainers and educators (in schools or companies) and the everyday practices of colleagues at work, which can significantly influence ‘young workers’. In addition, studies (N = 3 (The small number of studies on the pandemic is probably attributed to the short time span. Studies are certainly underway, and publications currently available on this subject are probably out of selection (press articles, magazines, and non-peer reviewed papers)), including two studies in Austria) highlight the impact of the Coronavirus Disease 2019 (COVID-19) pandemic, noting a significant increase in depressive symptoms, anxiety, eating, and sleep disorders among young people in training,^20^ skin irritation among nursing apprentices,^21^ and an increase in alcohol and cannabis use among this population.^22^

### OHS in context: organisational and social aspects

After a first section offering a description of the literature identified, this section provides a narrative analysis. Numerous studies agree that social and organisational aspects, like workplace characteristics, constraints and social interactions, have a greater impact on ‘young workers’ OHS in comparison than age or other individual factors.^9,23^ The aim of this section is to focus on studies that highlight the link between OHS and these aspects, focusing on how they have been addressed to date. Three aspects of the OHS of ‘young workers’ that have recently been explored in the literature are addressed here: the organisational constraints, the injuries considered through employment status and differentiated damages related to gender.

#### Organisational constraints

While the characteristics and constraints of work have rarely been considered in studies prior to 2005, as pointed out by Laberge and Ledoux,^9^ the literature of the last fifteen years has made greater efforts to do so, and nearly half of them include this dimension (N = 105). Most studies refer to or integrate variables derived from the Karasek and Theorell^24^ model, which identifies three vectors of pathogenic stress (depression and anxiety): high work demands placed on the worker, low decision latitude (i.e., the discretion given to the worker in deciding how to meet these demands), and lack of social support. The combination of these three factors strongly affects health pathways.^25^ However, the literature mainly focuses on one or two stress factors and their correlation with mental health impairment or accidents. Indeed, the studies mainly focus on social support (N = 53), likely due to the emphasis on the juvenile population, which is often view in relation to its family and social environment. In contrast, there is less focus on work demands (N = 27) and decision latitude (N = 16).

##### Social support

Social support refers mainly to socio-emotional and technical support from colleagues and supervisors, but also from trade unions and the entourage (family, friends, etc.). The literature addresses this question in relation to one of the following three aspects: the relations between low social support and psychological distress, the effects of low social support on the occurrence of work-related accidents, and the effects of the integration of trade unions in a company on injury reporting or on the way ‘young workers’ think about OHS issues.

A first body of work focuses on the link between low social support – generated by relational difficulties at work or a lack of supervision – and psychological distress. This impact is generally measured by levels of anxiety and depression.^26–28^ Low social support can result in harassment, teasing, or aggression from customers,^29,30^ as well as repetitive tasks and poor skills transfer, beyond simply productive tasks.^31^ This can lead to feelings of low self-esteem and distress,^32^ and to drug or alcohol use.^32,33^ Organisational factors, such as negative behaviours that are tolerated in the work environment, contribute to the lack of social support.^4^ Conversely, other research investigates the different sources of social support that have a protective effect against psychological distress^34–38^: formal support systems involving trainers or mentors and informal support from peers, colleagues, and relatives.^39^ For apprentices, these various forms of support are among the main criteria for a positive assessment of their training.^40^ In some cases, they promote professional integration and reduce stress and anxiety factors.^41^

A second body of work highlights the strong correlation between poor social support, particularly a lack of supervision, and the occurrence of work-related accidents, especially in hazardous operations.^18,42,43^ Social support, on the one hand, is a protective factor against harassment, which tends to occur when people are isolated. Victims of harassment, often caught in a dynamic of escalating violence,^30^ are more reluctant to seek help when carrying out risky tasks.^4^ This reluctance to ask for help increases their risk of accidents. Although harassment is often overlooked in research on the OHS of ‘young workers’,^44^ it is an important factor in accident occurrence. On the other hand, support from supervisors and the work collective encourages apprentices to seek assistance when experiencing difficulties in learning or performing certain tasks.^45,46^ This support helps them become aware of risks, learn OHS skills in practice,^47^ and refuse to perform tasks that are too dangerous.^48^

A third body of work focuses on the role of trade unions in OHS issues for ‘young workers’ (prevention, knowledge of rights and obligations, etc.).^49–54^ A study on apprentices in the construction industry established a correlation between the degree of closeness to the trade union organisation and the number of reports of injury (neck pain, back pain, and MSDs).^55^ In addition, apprentices who claim to be in contact with a trade union are more integrated into the work collective and therefore have stronger social support. They are also more willing and able to identify and control occupational health hazards and to adapt the way they carry out their tasks to prevent pain.^55^

##### Work demands and decision latitude

Some of the literature refer to Karasek's initial model^56^: a high work demands combined with low decision latitude leads to job strain, which affects well-being at work. The high work demands on ‘young workers’ is considered to be a factor that heavily affects their OHS (e.g.,^57^), but it varies widely depending on the sector^51,53,58,59^ and the country.^51^ High work demands is frequently linked to multiple jobs, a common phenomenon among ‘young workers’ who hold several jobs at the same time. It may also be related to the specific working conditions of this population, who are forced to work irregular hours or long periods without extended rest.^33,34,58^ However, the situation of apprentices differs in the sense that they have a lower work demands than adult workers, but less job decision latitude.^51,58,60^ The literature also indicates that a ‘limited margin of manoeuvre forces the worker to adopt safety strategies, particularly individual ones, that can be costly for mental and physical health or productivity’ (^47^ p. 251). Finally, some studies make a relation between limited decision latitude of ‘young workers’ and the rate of accidents at work,^43^ sickness absence,^34^ and declining mental health.^25,38^

Although the attention paid to the characteristics and constraints of work that Laberge and Ledoux (2011) had hoped for has largely developed, it should be emphasised that sociological approaches are still a minority. The organisation and division of labour and the social relationships between workers are rarely analysed. As a result, high work demands, room for manoeuvre and even social support are seldom considered from the perspective of employment status.

#### Social factors influencing health damage

In most studies on OHS of ‘young workers’, the age category is the primary focus. As a result, this population has been homogenised, which deeply impacted public policies and prevention campaigns.^61^ Another consequence has been an essentialist view of the relationship between health and young people, whose vulnerability and high accident rates have been attributed to their low awareness of work dangers, their taste for risk-taking and their lower cognitive maturity.^62,63^ However, an increasing number of studies are examining OHS injuries differentiated by sector, employment status, and gender. This section looks in more detail at the two latter.^51,61^

##### Damage to health related to employment status

The influence of the various employment statuses of ‘young workers’ (skilled or unskilled workers, young people in training) on OHS has been noted by several authors.^5^ First, it influences the working and training conditions, tasks performed, degree of responsibility, and working hours (nights, weekends, etc.). Second, some of the conditions ‘young workers’ are often assigned to, directly affect health, as temporary contracts,^51^ part-time work, frequent changes, multiple jobs,^64^ and atypical working hours.^51^

By way of illustration, a study of young sales workers shows that skilled workers, apprentice, sabbatical workers, student workers, or school leavers are exposed to vastly different risks. The working conditions to which ‘young workers’ are exposed (work time, working hours, and type of work) and their attitude to risk (fatalism, banalisation, caution, conformism, etc.) are strongly associated with their status.^61^ Although they regularly take unconsidered risks to meet the expectations of the trainer (manager), apprentices associate risk-taking with the attitudes of young unskilled workers. For their part, young unskilled workers (school leavers) assigned to subordinate handling jobs and repetitive tasks adopt an attitude of resignation towards risks and physical hardship, which are suffered rather than mastered or controllable.

If we compare apprentices with other youth populations, the results seem ambiguous, even contradictory. On the one hand, they appear to benefit from longer periods of initiation and peer training than young unskilled workers,^23^ their status therefore constitutes a protective factor. On the other hand, they appear to be four times more likely to suffer an accident than young people in employment.^42^ In this case, their status as young people in training appears to be an aggravating factor.

##### Gender-related health damage

The relationship between gender and health damage is complex. Indeed, quantitative^65^ and statistical^51,66,67^ studies show that, proportionally, young men have a higher rate of occupational accidents than young women. Other studies indicate that young women are more affected by certain risks, such as stress or MSDs,^53,68^ and are much more likely to be victims of harassment.^51,69^ However, a previous literature review states that gender is not associated with injury ‘when job characteristics are controlled’.^6^ As a result, according to Breslin et al.,^69^ several studies that count and weight the occurrence of accidents by gender in certain occupational sectors, such as sales, find no significant difference between young men and young women. Gender-differentiated harm appears then to be linked mainly to the unequal distribution of men and women in different sectors or occupations,^6,42^ which results from the sexual division of labour.

In this perspective, there are still few studies that ‘have examined in depth how individuals experience risks in the workplace, and how these experiences are gendered’ (^69^ p. 784). Some studies show that the service and health care, which are largely feminised, involve not only strong relational constraints linked to a confrontation with clients or patients but also postural constraints or the repetition of gestures.^52,70^ The sexual division of labour also explains the higher proportion of young women in traditionally male sectors who suffer mental health impairments.^52,70^ Canadian studies on these female pioneers, show, particularly through interviews, that their mental health can be adversely affected by repeated hostile reception, sexist jokes, denigration, and sexual harassment both at school and at the training company.^70,71^ In addition, these pioneering women, who have minimal legitimacy in these ‘male’ occupations,^70^ are tested by work collectives hostile to their presence.^72^ As seen above, harassment and poor integration contribute to isolating individuals, who lack group support and collective defensive strategies and are reluctant to ask for help.

A final aspect relates to gendered socialisation, which shapes differentiated representations of OHS. In feminised sectors, such as hairdressing, vocational students see OHS primarily in terms of protecting their customers’ health^70^ and consider their personal health to be their responsibility, as their training encourages them to do.^52^ In male-dominated sectors, there is a tendency to trivialise injuries and pain, with ‘young workers’ avoiding complaining about injuries, consistent with the virile culture of the occupation or to mark their belonging to the adult work collective. While a form of banalisation also exists among young women, in particular so as not to inconvenience customers,^73^ their complaints and concerns tend to be systematically ignored, discredited, or muted by their colleagues and hierarchy.^69^

## Discussion and conclusion

### Overview of the main topics of the systematic review

The systematic review presented in the first section of the results leads to some observations.

The first observation is the heterogeneity of the populations investigated under the same designation. Thus, behind ‘young workers’ various situations can be found: individuals working with or without qualifications and those in training. The latter can be apprentices, that is to say salaried employees following alternating training (apprentices) or students on full-time VET school (vocational students). These two populations are confronted with distinct training environments (practice workrooms *vs*. productive work situations) and constraints (pressure for school results *vs*. pressure of the productive logic). The difficulty in isolating the specific population of apprentices undoubtedly explains the scarcity of analyses that focus on their status and on the specific training and working conditions associated with it. In particular, the specific constraints of dual VET, such as the tension between producing and training.^74^

A second observation concerns the primacy of medical research, which favours epidemiological approaches and quantitative methods. Prioritising the identification of risk factors, particularly individual factors (sex, age, physical characteristics, and pre-existing state of health), leads to an individualistic conception of health, to the detriment of organisational and social issues. Studies from other disciplines, particularly social sciences, which opt to use qualitative and mixed methods, are rarer, despite a significant increase. This represents 10 (5%) of the 189 articles investigated by Laberge and Ledoux^9^ versus 36 (16%, qualitative) or even 63 (30%, mixed) of the 193 articles of the present corpus. This important evolution makes it possible to take other factors into consideration: the organisation of work, working conditions, the social division of labour, social relations, particularly those of class, gender or race.

Third observation, the entry by the category of age is now commonplace. This approach is based on the physical and mental developmental stage of ‘young workers’ as risk factors. Age is however rarely linked to an analysis of working conditions or employment status, as Laberge and Ledoux^9^ called for. On the contrary, it is mainly used as an essentialising category to explain many of the injuries by ‘adolescence’ alone. The focus is then on the strong propensity of adolescents to take risks or their weaker capacity to measure the consequences of exposure, and not on social constraints, the power dynamics at work, and on the vulnerabilities associated with the ‘young workers’ status, including the apprentice.

Fourth, physical health through work-related accidents (40%) and other physical injuries (28%) are still dominant. This can be understood as a response to the concerns of public authorities^1^ faced with the over-accidentability of ‘young workers’, identified by national statistics, and the related costs. However, mental health is occupying an increasing place, with a third of the literature reviewed devoted to it (33%), in comparison with the low rate pointed out by Laberge and Ledoux^9^ (10%) for the previous period. This steady increase reflects, among other things, the deterioration in young people's health highlighted more generally in recent statistics.^75^ In addition, the share of mental health in occupational health costs has become a matter of public concern, particularly as regards the juvenile population.

Different evolutions have been observed since the literature review of Laberge and Ledoux.^9^ They make it possible to deepen knowledge on OHS of ‘young workers’, and by extension on apprentices.

### Overview of promising themes identified through the narrative analysis

The second section of the results has focused on some themes that we consider central to conduct the further analyses mentioned here above.

It is noteworthy that the analysis of organisational constraints proposed in the studies reviewed rarely integrate the three stress vectors of the Karasek and Theorell^24^ model. Indeed, they focus mainly on social support (N = 53), less on work demands (N = 27) and decision latitude (N = 16). The emphasis on social support is probably related to the juvenile population studied, which is frequently considered through its family and social environment. Work demands is the second aspect addressed by these studies, mainly through its links with health risks, particularly when there is little decision latitude. It is also noted that apprentices tend to have a lower work demands than other workers, but that their situations are very heterogeneous depending on sectors and countries. Finally, the studies focusing on decision latitude emphasise the fact that ‘young workers’, including apprentices, have a low decision latitude. It is noteworthy that work demands, decision latitude and even social support are seldom considered from the perspective of employment status.

Although the attention paid to the characteristics and constraints of work that Laberge and Ledoux had hoped for has largely developed, it should be emphasised that psychological models (e.g., 24) are dominant. Even if these studies take into account the organisational constraints, the analyses of working conditions, in terms of organisation and division of labour, of social relationships between workers or of employment statuses (young worker, novice, and apprentice) are rare. Sociological approaches are still a minority.

However, even if rare, analyses of OHS from the point of view of employment status is proving effective. The different statuses highlight specific working conditions, which have more or less direct repercussions in terms of health. In the case of apprentices, their status can be both a protective and an aggravating factor, this complexity merits further analysis.

In the same vein, analyses in terms of gender are interesting as long as we do not seek to make a simple comparison between men and women. Sectors of activity in which women and men work have to be further analysed, in order to identify the specific problems they face, in terms of constraints, working conditions, arduousness and also occupational cultures.

The second part of results highlights the interest of adopting more sociological perspectives and using qualitative methods, in order to identify the complexity of working and training conditions of apprentices, the specific constraints of dual VET or the ambiguity of apprentice's status, both protective and at risk.

### Perspectives on research

The findings have highlighted the relevance of considering employment status. Following the discussion, it seems essential to pursue this line of research, in particular to gain a better understanding of the OHS issues faced by apprentices.

In fact, entry by status involves distinguishing between the different situations made invisible by the heterogeneous category of ‘young workers’. This allows to highlight the working conditions and constraints specific to the different statuses – salaried or not, qualified or not, in training, and, in certain cases, to question the training conditions, formal or not, addressed to these different populations. Referring to employment status as a starting point sheds also light on the analysis of certain constraints (work demand, decision latitude, relational issues) as being the result of power relationships in terms of positions occupied in the social division of labour and in the various social relations, and not solely as a consequence of age. This sociological approach considers the occupational health of these different types of ‘young workers’ through the intersecting issues of work and employment. Far from the dominant analyses which tend to individualise, or even essentialise, OHS issues, looking at employment status points out that this question remains fundamentally linked to the protective or pathogenic context in which work takes place.

This perspective can help us to better understand the circumstances in which ‘young workers’ are placed in situations likely to be detrimental to their health or are exposed *nolens volens* to risks, despite the formal regulations on health protection in the workplace. In the case of apprentices, their status is both a protective and an aggravating factor^23,42^ when it comes to occupational health. It is determined by an intermediate position between students and workers, between trainees and production employees, but also by a particularly subordinate position in the organisation and division of work. The specific features of the apprentice status imply taking into account both work and training systems, and their constraints, such as the tension between producing and training.^74^

This research can also lead to courses of action that enable effective systems to be implemented to protect the mental and physical health of apprentices, taking into account the social relations at work, the issues specific to training in a production context, and the obstacles preventing the transmission of health knowledge, defensive strategies and, simultaneously, their appropriation by the groups concerned.
